# Effusion cytology of EBV-associated lymphoma: a concise review

**DOI:** 10.1007/s44313-025-00088-0

**Published:** 2025-07-02

**Authors:** Chih-Yi Liu, Yen-Chuan Hsieh, Sheng-Tsung Chang, Hung-Chang Wu, Shang-Wen Chen, Shih-Sung Chuang

**Affiliations:** 1 Department of Pathology, Zhongli District, Ten Chan Hospital, Taoyuan City, Taiwan; 2 School of Medicine, National Tsing Hua University, Hsinchu City, Taiwan; 3 Department of Clinical Pathology, Chi Mei Medical Center, Tainan City, Taiwan; 4 Department of Pathology, Chi Mei Medical Center, Tainan City, Taiwan; 5 Department of Nursing, National Tainan Institute of Nursing, Tainan City, Taiwan; 6 Department of Internal Medicine, Chi Mei Medical Center, Tainan City, Taiwan; 7 Division of Hemato-Oncology, Department of Internal Medicine, Chi Mei Medical Center, Liouying, Tainan, Taiwan

**Keywords:** Azurophilic granules, Cytotoxic phenotype, EBER, EBV, Effusion cytology, Immunophenotyping, T/NK-cell lymphoma

## Abstract

Epstein-Barr virus (EBV)-associated lymphomas can, on rare occasions, involve body cavities, making effusion cytology an important diagnostic tool. This mini-review explores the spectrum of EBV-related lymphomas that may be detected in serous fluids, including EBV-positive nodal T/NK-cell lymphoma (EBV + nT/NKCL), extranodal NK/T-cell lymphoma, primary effusion lymphoma, EBV-positive diffuse large B-cell lymphoma, and classic Hodgkin lymphoma. We present an index case of EBV + nT/NKCL with lymphomatous pleural effusion and discuss the cytologic features, differential diagnoses, and role of ancillary studies such as immunocytochemistry, EBER in situ hybridization, and molecular assays. Accurate diagnosis requires the integration of cytomorphologic, immunophenotypic, and molecular findings with clinical information to establish a definitive diagnosis and distinguish these aggressive lymphomas from reactive and non-hematologic mimics.

## Introduction

Epstein-Barr virus (EBV) is a ubiquitous gamma herpesvirus implicated in a variety of lymphoproliferative disorders, particularly among immunocompromised individuals and the elderly. EBV-associated lymphomas encompass a wide spectrum, including classic Hodgkin lymphoma (CHL), B-cell, and NK/T-cell lymphomas. The 5th edition of the WHO Classification of Haematolymphoid Tumours (WHO-HAEM5) recognizes two specific EBV-positive T-cell and NK-cell lymphoma entities: extranodal NK/T-cell lymphoma (ENKTL) and EBV-positive nodal T- and NK-cell lymphoma (EBV + nT/NKCL) [1]. The cytological features of these two lymphomas in body cavity effusions remain under-characterized. Nonetheless, effusion cytology may serve as a valuable tool for the rapid and minimally invasive diagnosis of such malignancies.

EBV + nT/NKCL is a newly recognized lymphoma entity in WHO-HAEM5 [1]. It is an aggressive, EBV-associated cytotoxic lymphoma of T- or NK-cell lineage, typically presenting with nodal involvement in older adults. Previously, it was referred to in the literature as nodal NK-like cytotoxic T-cell lymphoma [1]. It is considered the nodal counterpart of ENKTL, which classically involves the upper aerodigestive tract [2]. We recently demonstrated that ENKTL in effusion specimens may display anaplastic large-cell morphology and azurophilic cytoplasmic granules [3]. However, the cytological features of EBV-related lymphomas in effusion samples have rarely been described. In this review, we present an index case of EBV + nT/NKCL with extensive pleural effusion and provide a comprehensive overview of the cytologic characteristics of various EBV-associated lymphomas in serous fluids.

### Report of the Index Case

A previously healthy 65-year-old woman presented with fever, abdominal fullness, and flank pain. Computed tomography (CT) revealed bilateral pleural effusions, retroperitoneal and iliac lymphadenopathy, ascites, and hepatic and gynecologic lesions. A para-aortic lymph node biopsy confirmed EBV + nT/NKCL. Cytologic analysis of pleural effusion and bone marrow confirmed involvement of both sites, indicating stage IV disease. Positron emission tomography (PET)-CT revealed disseminated disease but no nasal or paranasal lesions, thereby excluding a diagnosis of ENKTL. Laboratory tests showed elevated lactate dehydrogenase (LDH) levels (819 IU/L) and a markedly elevated EBV viral load (3,059,277 IU/mL). The patient’s International Prognostic Index (IPI) score was 5. Due to the rapid disease progression, she received only palliative care without chemotherapy and died within 2 weeks.

**Fig. 1:**
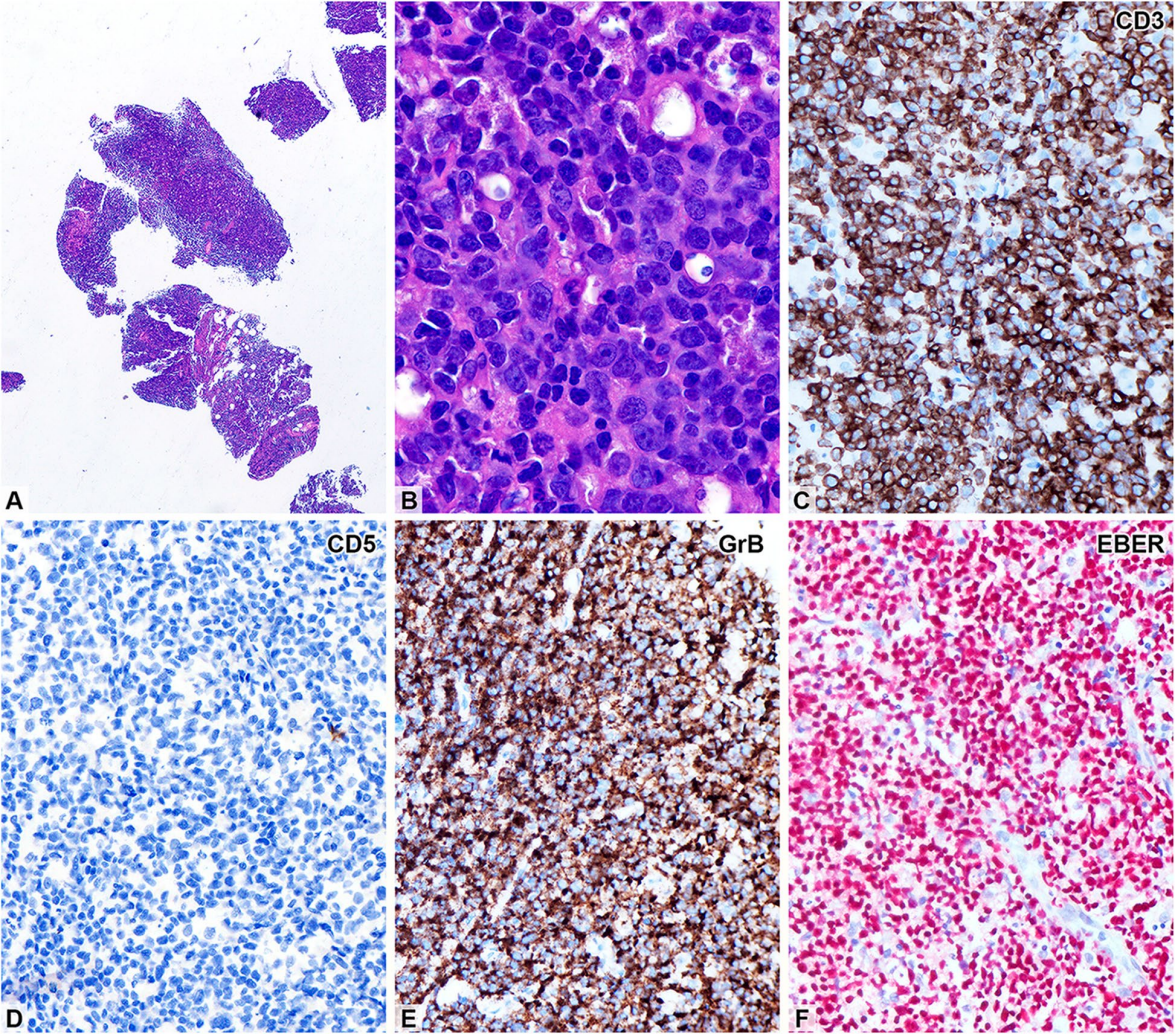
Needle biopsy from a para-aortic lymph node. (**A**) Fragmented lymphoid tissue cores showing diffuse infiltration (H&E, × 40). (**B**) Sheets of large, atypical lymphocytes with vesicular nuclei, some with irregular nuclear contours and small nucleoli. Immunohistochemically, the tumor cells express CD2, CD3 (2**C**), TIA-1, and granzyme B (2**E**, GrB), but not CD4, CD5 (2**D**), CD8, CD20, CD138, TCR-BF1, or TCR-delta. (**F**) The tumor cells are diffusely positive for EBV by in situ hybridization (**C**-**F**, × 400)

Needle core biopsy of the para-aortic node revealed diffuse atypical lymphocytic infiltration with focal tumor necrosis. The tumor cells were admixed with abundant karyorrhectic debris, which was phagocytosed by benign histiocytes, creating a starry-sky pattern. The atypical lymphocytes were medium to large in size, with irregular nuclear contours, small nucleoli, and frequent mitoses (Fig. 1). Immunohistochemically, these cells expressed CD2, CD3, T-cell intracellular antigen (TIA)−1, and granzyme B, but were negative for CD4, CD5, CD8, CD20, CD138, T-cell receptor (TCR)-betaF1, and TCR-delta. The Ki-67 proliferation index was approximately 80%. In situ hybridization for EBV-encoded RNA (EBER) was diffusely positive. Polymerase chain reaction (PCR)-based clonality testing for TCR-gamma (*TRG*) gene rearrangement showed polyclonal results using both in-house primers and BIOMED-2 protocols, as previously described [4, 5]. These findings suggest that this case of EBV + nT/NKCL was likely of NK-cell rather than T-cell lineage.

**Fig. 2:**
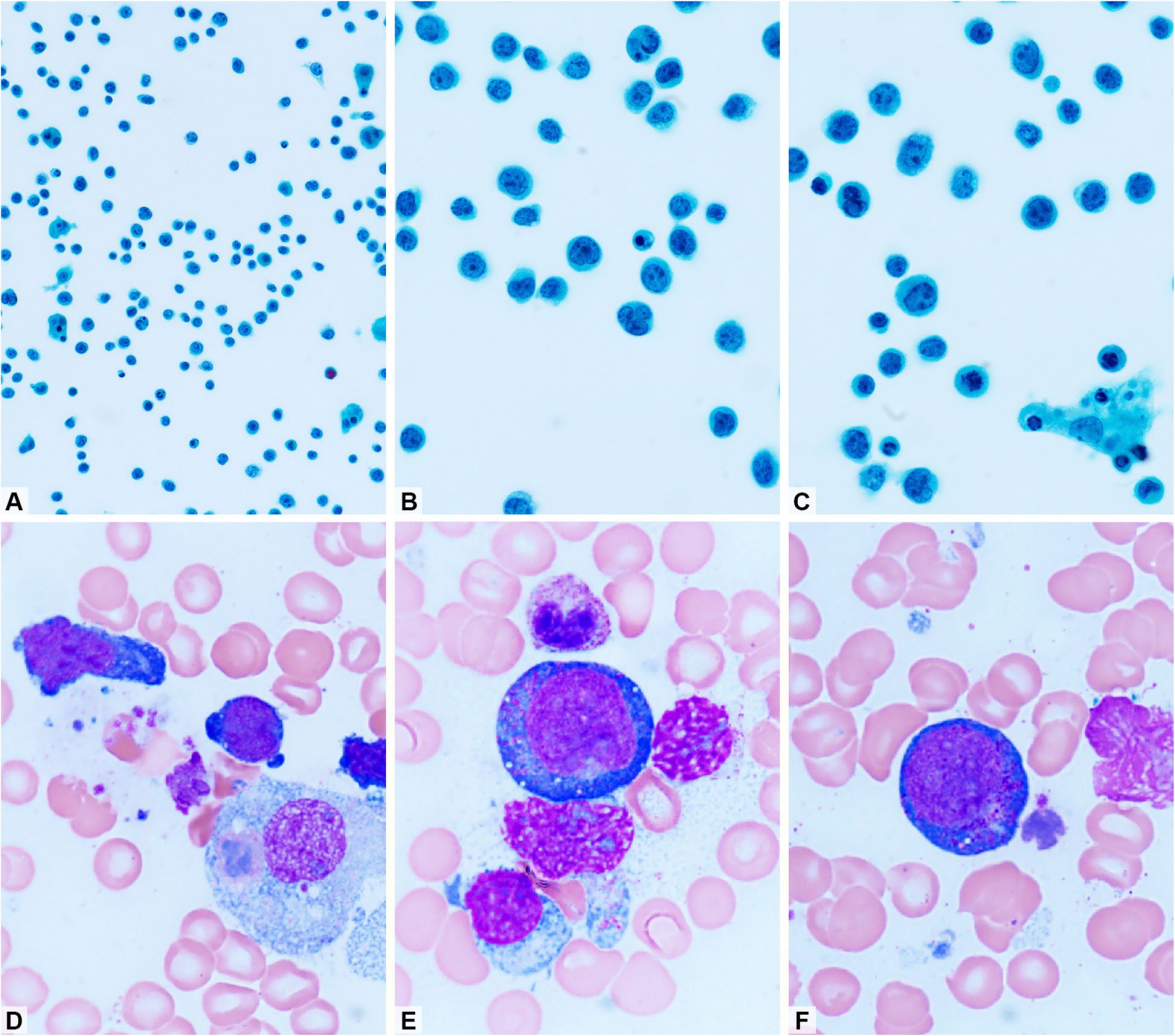
Pleural effusion. (**A**) The effusion specimen is cellular with non-cohesive cells. (**B**, **C**) Singly scattered medium- to large-sized cells with vesicular nuclei, some indented, and one to three small nucleoli (**A**–**C**, Papanicolaou stain; **A**, × 400; **B** and **C**, × 1,000). (**D**–**F**) Tumor cells in the bone marrow aspirate smear (Liu stain; close-up view from × 1,000). (**D**) Two tumor cells and a benign histiocyte with hemophagocytosis. (**E**, **F**) Lymphoma cells show vesicular nuclei, small nucleoli, and a few azurophilic cytoplasmic granules

Pleural fluid was collected via thoracentesis. Papanicolaou-stained cytology smears revealed large atypical lymphocytes with slightly irregular nuclear contours and moderate amounts of cytoplasm (Fig. 2A-2C). Bone marrow aspirate stained with Liu's stain showed cells with prominent azurophilic cytoplasmic granules (Fig. 2D-2F).

This case demonstrates that EBV + nT/NKCL may rarely present with effusions, posing a diagnostic challenge that necessitates thorough cytological evaluation [2, 6, 7]. However, diagnosis should not be based solely on cytological findings, as distinguishing this entity from ENKTL requires PET-CT imaging and biopsy of the upper aerodigestive tract [2].

### Cytologic Features of EBV-Related Lymphomas in Effusions

Effusion cytology is a valuable diagnostic tool in the evaluation of EBV-associated lymphomas. Recognition of characteristic features, such as cell size, the presence of cytoplasmic granules, and immunophenotypic profiles, provides critical diagnostic clues. Accurate diagnosis requires the integration of cytomorphologic assessment with immunophenotyping and, in some cases, molecular data and clinical context.

#### *EBV-positive nodal T/NK-cell lymphoma (EBV* + *nT/NKCL)*

EBV + nT/NKCL is more prevalent in Asia than in Western countries and typically presents with nodal involvement in the absence of nasal lesions, a key feature that distinguishes it from ENKTL [2]. Ohshima K. et al. characterized the tumor cells as having a cytotoxic phenotype of either T- or NK-cell origin [7] and highlighted the presence of cytoplasmic azurophilic granules in imprint cytology. Tao J. et al. reported a rare case of this lymphoma in a West African male, describing large atypical lymphocytes with markedly irregular nuclei and basophilic cytoplasm, some containing azurophilic granules [6].

EBV + nT/NKCL is typically composed of medium to large atypical lymphoid cells with vesicular chromatin, irregular nuclear contours, and small but distinct nucleoli [8]. A hallmark feature is the presence of azurophilic cytoplasmic granules, which are detectable only with Romanowsky-type stains (e.g., Liu and Giemsa) but not with Papanicolaou stain [3, 9]. These granules indicate a cytotoxic phenotype and, although not pathognomonic, are highly suggestive when seen in conjunction with a characteristic immunophenotypic profile (CD2 +, CD3 +, TIA-1 +, granzyme B +, EBER +). Notably, these cells are often negative for CD4 and CD5. The majority of cases are of T-cell origin, as evidenced by TCR antigen expression and/or clonal TCR gene rearrangement. A minority are of NK-cell origin, characterized by absent TCR expression and polyclonal TCR gene rearrangement, as demonstrated in our index case [1].

#### Extranodal NK/T-Cell Lymphoma (ENKTL)

Cytologically, effusions from ENKTL may resemble those of EBV + nT/NKCL but often show more pronounced anaplastic large cell morphology, characterized by coarse chromatin and azurophilic cytoplasmic granules [3, 8, 10]. The cytologic background in ENKTL is frequently necrotic, with abundant karyorrhectic debris. CD56 expression is commonly seen in ENKTL but is typically absent in EBV + nT/NKCL [1, 2]. Clinical presentation, particularly involvement of the upper aerodigestive tract, including the nasal cavity and paranasal sinuses, often provides a key diagnostic clue [3, 8, 9, 11].

The presence of azurophilic cytoplasmic granules—best visualized with Romanowsky-type stains such as Liu, Giemsa, and Wright—serves as a cytologic hallmark of cytotoxic T- or NK-cell derivation [3, 9, 11, 12]. Uniformly sized azurophilic granules are seen in large granular lymphocytes (LGLs) on peripheral blood smears. LGLs may be either CD8-positive cytotoxic T cells or CD56-positive NK cells. The leukemic counterparts of LGLs—T-cell or NK-cell LGL leukemias—typically exhibit pleomorphic nuclei and azurophilic granules. Similarly, in malignant lymphomas, tumor cells with a cytotoxic phenotype may represent either cytotoxic T-cell or NK-cell lymphoma. For example, the neoplastic cells in monomorphic epitheliotropic intestinal T-cell lymphoma and ENKTL, both cytotoxic lymphomas, show characteristic azurophilic granules [3, 9, 11–13]. Therefore, morphological evaluation alone is insufficient for a definitive diagnosis and must be integrated with clinical and imaging findings.

#### EBV-Positive Diffuse Large B-Cell Lymphoma (DLBCL)

In EBV-positive DLBCL, effusion samples typically contain large immunoblastic cells with a high mitotic index and occasional plasmacytoid differentiation. These effusions often show large, pleomorphic lymphoid cells with centroblastic or immunoblastic morphology, prominent nucleoli, and basophilic cytoplasm [14, 15]. Although cytologically less pleomorphic than primary effusion lymphoma (PEL) or ENKTL, EBV-positive DLBCL must be differentiated from plasmablastic lymphoma, which also exhibits abundant basophilic cytoplasm and prominent nucleoli but is not consistently associated with EBV. The neoplastic cells in EBV-positive DLBCL express CD20, CD79a, and MUM1, and are strongly EBER-positive. Immunophenotyping for CD20, MUM1, and EBER is essential for diagnosis [16].

#### Classic Hodgkin Lymphoma (CHL)

Effusions associated with EBV-positive CHL are rare but pose a diagnostic challenge when present. In serous fluids, lymphocytes typically predominate, although scattered to rare Reed-Sternberg (RS) cells may be observed [15]. The identification of RS cells within a reactive background of lymphocytes, eosinophils, and histiocytes is a key diagnostic feature [15, 17]. A definitive diagnosis requires the recognition and confirmation of RS cells by immunophenotyping or histologic examination of tissue sections. In EBV-associated cases, RS cells typically express CD30, CD15, and PAX5, and are EBER-positive. However, reactive mesothelial hyperplasia or anaplastic large cell lymphoma can present with overlapping cytologic features, necessitating careful immunophenotypic evaluation.

#### Primary Effusion Lymphoma (PEL)

**Fig. 3:**
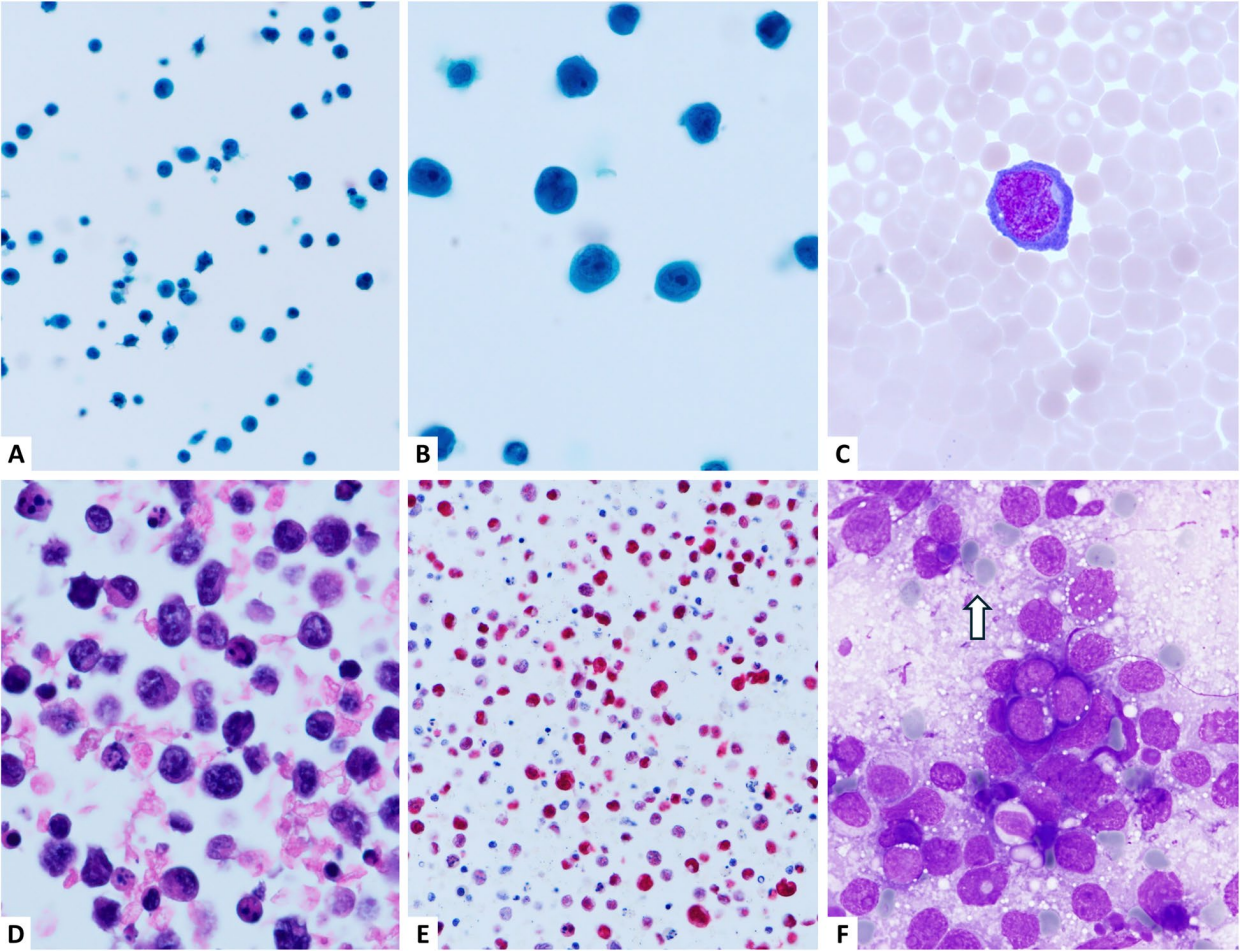
An example of HIV-negative primary effusion lymphoma from a 78-year-old man who presented with massive right pleural effusion (Panels A–E). He was negative for HIV infection. (**A**, **B**) Papanicolaou stain at × 400 and × 1,000 magnification, respectively, showing large neoplastic cells with vesicular nuclei, some containing a prominent central nucleolus. (**C**) Wright-Giemsa stain shows a neoplastic cell with plasmablastic features and a perinuclear hof (× 1,000). (**D**) Cell block section reveals large, non-cohesive tumor cells (H&E stain, × 1,000). (**E**) The tumor cells are positive for EBV by in situ hybridization (× 400). (**F**) An example of Burkitt lymphoma for comparison. Imprint cytology with Liu stain shows monomorphic neoplastic cells with round nuclei and small cytoplasmic lipid vacuoles (× 1,000). Lymphoglandular bodies are also present (arrow)

PEL typically presents as a lymphomatous effusion without associated mass lesions. It poses a unique diagnostic challenge in effusion cytology (Fig. 3). The malignant cells are large, pleomorphic lymphoid cells with prominent nucleoli and vacuolated cytoplasm. These cells often lack cohesion and appear within a hemorrhagic background, mimicking high-grade non-Hodgkin lymphomas or even metastatic carcinoma [16–18]. Co-infection with HHV8 (and often EBV) is a defining feature. Diagnosis relies on positive immunostaining for CD138 and HHV8 latency-associated nuclear antigen. The immunophenotypic profile typically includes CD45 +, CD30 ±, CD138 +, HHV-8 +, and EBER +. The absence of conventional B-cell markers and surface immunoglobulin expression may further complicate the diagnosis. This underscores the importance of interpreting cytologic findings within the clinical context, particularly the patient’s HIV status [16–18].

#### Burkitt lymphoma (BL)

Burkitt lymphoma is an aggressive B-cell neoplasm frequently associated with EBV in endemic regions and in immunodeficiency-related settings. The prevalence of EBV positivity in sporadic Burkitt lymphoma (BL) varies by geographic region and across population-based studies. For example, a study from Taiwan reported EBV positivity in approximately 20% of sporadic BL cases [19]. Although involvement of serous effusions is uncommon, cytologic evaluation can support the diagnosis, when present. Cytologically, BL is characterized by a monomorphic population of medium-sized lymphoid cells with a high nuclear-to-cytoplasmic (N/C) ratio. The nuclei are round with coarse chromatin and multiple small basophilic nucleoli. The cytoplasm is deeply basophilic and often contains multiple small cytoplasmic lipid vacuoles, which are particularly evident with Romanowsky-type stains (e.g., Giemsa and Liu) (Fig. 3F) [20, 21]. The background typically displays a “starry-sky” pattern due to the presence of scattered tingible-body macrophages and numerous apoptotic bodies, reflecting the tumor's high proliferative activity [22]. Immunophenotypically, the neoplastic cells in BL express CD20, CD10, and BCL-6, and exhibit a very high Ki-67 proliferation index (> 95%). EBER is positive in most endemic and HIV-associated cases. Distinguishing BL from other high-grade B-cell neoplasms and lymphoblastic lymphoma requires immunophenotyping and cytogenetic analysis, particularly the detection of *MYC* gene rearrangements [20, 21, 23].

#### Plasmablastic lymphoma (PBL)

Plasmablastic lymphoma is a rare and aggressive lymphoma with plasmacytic differentiation, strongly associated with HIV infection and EBV. Although it primarily arises in extranodal sites, such as the oral cavity and gastrointestinal tract, pleural or peritoneal effusion involvement has been documented, particularly in immunocompromised individuals [24–26]. Cytologically, PBL in effusion specimens shows large atypical cells with eccentrically placed nuclei, open chromatin, and prominent nucleoli. The cytoplasm is moderate to abundant, deeply basophilic, and may display perinuclear clearing or a paranuclear hof. The neoplastic cells may exhibit plasmacytoid, immunoblastic, or anaplastic features. Multinucleation and brisk mitotic activity are common findings [24, 27]. The background may be necrotic or inflammatory, with scattered apoptotic debris [27]. Immunocytochemistry reveals negativity for pan-B-cell markers (CD20and PAX5) and positivity for plasma cell markers such as CD138, CD38, and MUM1. Most cases are EBV-positive by EBER in situ hybridization [24–26]. Clinical context and immunophenotyping are essential for confirmation.

Table 1 lists the key cytologic and immunophenotypic features of various EBV-related lymphomas in serous effusions. EBV + nT/NKCL is typically composed of medium to large, atypical cells with azurophilic cytoplasmic granules and minimal background inflammation, and expresses cytotoxic markers including CD2, cytoplasmic CD3ε, TIA-1, and granzyme B, with EBER positivity. ENKTL shows more pleomorphic, anaplastic cells in a necrotic, debris-laden background, with CD56 positivity serving as a key differentiator. PEL is characterized by large plasmablastic or immunoblastic cells in a hemorrhagic background, vacuolated cytoplasm, and positivity for CD138 and HHV8, often with co-existing EBV infection. EBV-positive DLBCL features immunoblastic or centroblastic morphology with abundant cytoplasm and necrosis, expressing CD20 and MUM1. CHL, although rare in effusions, displays characteristic Reed-Sternberg cells with amphophilic cytoplasm amidst a mixed inflammatory background, expressing CD30, CD15, and PAX5. BL may appear in effusions as a monomorphic population of medium-sized cells with round nuclei, coarse chromatin, multiple basophilic nucleoli, and deeply basophilic cytoplasm often containing lipid vacuoles; the cells express CD10, BCL6, and a nearly 100% Ki-67 index. PBL is composed of large plasmablast-like cells with eccentrically placed nuclei, prominent nucleoli, and basophilic cytoplasm, typically lacking CD20 but expressing plasma cell markers such as CD138, MUM1, and CD38. The consistent presence of EBER positivity across all entities highlights the central role of EBV in their pathogenesis, while the distinct cytologic and immunophenotypic profiles facilitate accurate classification.

**Table 1:** Cytologic Features of EBV-Related Lymphomas in Effusions

Lymphoma Subtype	Cell Morphology	Cytoplasmic Features	Background	Key Immunomarkers
EBV + nT/NKCL	Medium–large atypical cells	Azurophilic granules	Scant inflammation	CD2 +, CD3 +, TIA-1 +, GrB +, EBER +
ENKTL	Pleomorphic, anaplastic cells	Azurophilic granules	Necrotic, karyorrhectic debris	CD3 +, CD5-, CD56 ±, TIA-1 +, GrB +, EBER +
PEL	Immunoblastic, plasmablastic	Vacuolated, abundant	Hemorrhagic	CD138 +, HHV8 ±, EBER ±
EBV + DLBCL	Immunoblastic/centroblastic	Abundant, basophilic	Necrotic debris	CD20 +, MUM1 +, EBER +
CHL	RS cells, large binucleated	Moderate, amphophilic	Mixed inflammatory	CD30 +, CD15 +, PAX5 +, EBER ±
BL	Medium-sized cells with round nuclei	Basophilic cytoplasm containing lipid vacuoles	Apoptotic cells	CD20 +, MYC +, EBER ±, Ki-67 proliferation index ~ 100%
PBL	Large cells with plasmablastic morphology	Moderate to abundant, basophilic	Necrotic, apoptotic cells	CD138 +, MUM1 +, CD20-, EBER ±

*Abbreviations: BL* Burkitt lymphoma, *CHL* classic Hodgkin lymphoma, *EBER* in situ hybridization for EBV-encoded mRNA, *EBV* + *DLBCL* EBV-positive diffuse large B-cell lymphoma, *EBV* + *nT/NKCL* EBV-positive nodal T and NK cell lymphoma, *ENKTL* extranodal NK/T-cell lymphoma, *GrB* granzyme B, *HHV8* human herpesvirus 8, *MUM1* multiple myeloma oncogene 1, *PAX5* paired box 5, *PBL* plasmablastic lymphoma, *PEL* primary effusion lymphoma, *RS* Reed-Sternberg, *TIA-1* T-cell intracellular antigen-1

### Differential Diagnosis

Cytologic evaluation of effusions in patients with EBV-related lymphomas provides a valuable diagnostic window into otherwise inaccessible disease sites, particularly in cases of advanced or disseminated disease. Among the spectrum of EBV-associated hematologic malignancies, effusion cytology reveals a broad range of morphologic phenotypes that require careful evaluation, immunophenotyping, and ancillary testing to establish a definitive diagnosis.

The differential diagnosis should first include reactive lymphocytosis, particularly in post-infectious or autoimmune conditions. These effusions typically display small, mature lymphocytes with smooth nuclear contours, dense chromatin, and an absence of cytoplasmic granules or mitotic Figs. [15]. Unlike lymphomas, reactive lymphocytes are polyclonal on T-cell receptor (TCR) gene rearrangement studies and negative for EBER by in situ hybridization [28].

Another important consideration is metastatic carcinoma, particularly adenocarcinoma, which may mimic lymphoma in effusion samples due to large cell size and a discohesive growth pattern. However, carcinoma cells often exhibit glandular arrangements, cytoplasmic vacuolation, and positive immunostaining for cytokeratins (e.g., AE1/AE3, CAM5.2), while lacking expression of lymphoid markers [15, 29]. Misclassification can lead to vastly different therapeutic strategies, emphasizing the importance of a comprehensive immunocytochemical panel for accurate diagnosis.

High-grade B-cell lymphomas, such as EBV-positive DLBCL and plasmablastic lymphoma (PBL), should also be included in the differential diagnosis, particularly when pleomorphic, immunoblastic morphology is observed. By definition, tumor cells in EBV-positive DLBCL are EBER-positive and typically express pan–B-cell antigens such as CD19 and CD20. They usually exhibit an activated B-cell-like immunophenotype, with expression of MUM1 [30], but are negative for plasmablastic markers such as CD138. PBL is a rare and aggressive lymphoma characterized by terminal B-cell differentiation, often expressing CD138 while lacking pan–B-cell markers [15, 17, 23, 24]. Around 50–70% of PBL cases are EBER-positive [26, 31]. In our recent study of nine PBL cases at a single institution in Taiwan, five patients (56%) presented with malignant effusions [24]. Interestingly, all five were EBV-negative; in contrast, three of the remaining four patients were EBER-positive. Notably, both EBV-positive DLBCL and PBL may occasionally present in effusions, but their tumor cells typically lack cytotoxic granules and are negative for T/NK-cell markers.

PEL, which features large plasmablastic cells within hemorrhagic effusions and no associated mass lesions, must also be considered. PEL cells express CD138 and MUM1 and are characteristically positive for HHV8, often with EBV co-infection. The absence of conventional B- or T-cell markers and the presence of HHV8 on immunohistochemistry are crucial for diagnosis [15, 16, 18].

EBV-related lymphomas in effusion cytology may also mimic neurogenic or other small round cell tumors, such as neuroblastoma, rhabdomyosarcoma, small cell carcinoma, and Merkel cell carcinoma. High-grade lymphomas are typically composed of medium to large atypical lymphoid cells with irregular nuclear contours, coarse chromatin, and lymphoglandular bodies, features readily observed with Romanowsky-type stains and less common in neurogenic tumors. In contrast, neuroblastoma presents with small round cells showing salt-and-pepper chromatin and often forms Homer Wright rosettes within a neuropil-like fibrillary background, which is not seen in lymphoid neoplasms [32]. Rhabdomyosarcoma may contain strap-like or tadpole-shaped cells with eccentric nuclei and eosinophilic cytoplasm; immunostaining for myogenin and desmin supports its identification [33]. Small cell carcinoma and Merkel cell carcinoma often form cohesive clusters in effusion cytology. In our previous study, we identified several cytologic features helpful in distinguishing Merkel cell carcinoma from lymphoma, including nuclear molding, Indian filing, and occasional pale cytoplasmic bodies [34]. Notably, lymphoglandular bodies were absent on Romanowsky-type stains [34]. These distinctive cytomorphologic features help narrow the differential diagnosis in effusion specimens. However, definitive diagnosis requires integration of cytologic findings with ancillary studies, including immunohistochemistry, in situ hybridization, and clonality assays, alongside clinical, radiologic, and laboratory data.

To navigate the complex differential diagnoses, ancillary studies are indispensable. EBER in situ hybridization remains the cornerstone for confirming EBV involvement. The pattern and intensity of EBER staining can offer valuable diagnostic clues: diffuse, strong positivity supports a diagnosis of lymphoma, whereas scattered or absent staining is more typical of reactive conditions. Flow cytometry is particularly useful in effusion samples with high cellularity, allowing for rapid and quantitative assessment of cell lineage (e.g., CD3, CD5, CD20, CD56), aberrant antigen expression, and monoclonal versus polytypic light chain expression.

Immunocytochemistry, performed on cell blocks or cytospin preparations, provides essential phenotypic context. Key markers for T/NK-cell differentiation include CD2, cytoplasmic CD3ε, CD5, CD56, TIA-1, granzyme B, and perforin. CD30 and ALK should be evaluated when anaplastic large cell lymphoma is considered in the differential. CD20, PAX5, and CD79a support B-cell lineage identification, while CD38, CD138, and EMA are useful for diagnosing plasmablastic lymphomas [15, 16, 18].

Molecular studies, particularly PCR-based TCR gene rearrangement analysis, are essential for confirming clonality in suspected T-cell neoplasms [28]. The combined use of in-house and standardized BIOMED-2 assays can improve sensitivity and confidence in interpreting clonality results [4, 5]. T-cell lymphomas typically show clonal TCR gene rearrangement, whereas NK-cell lymphomas are generally polyclonal. The absence of TCR antigen expression by immunohistochemistry—including TCR-betaF1, TCR-gamma, or TCR-delta, along with polyclonal TCR gene rearrangement, supports an NK-cell lineage.

Lastly, cytochemical staining plays a critical, though often underappreciated, role. While Papanicolaou stain provides excellent nuclear detail, Romanowsky-type stains (e.g., Liu, Giemsa, Wright) are uniquely suited to reveal cytoplasmic azurophilic granules. Their presence strongly suggests a cytotoxic phenotype and, when interpreted in conjunction with clinical, immunophenotypic, and molecular findings, helps distinguish aggressive EBV-related lymphomas from benign or non-lymphoid processes.

## Conclusions

In summary, the evaluation of EBV-related lymphomas in effusions requires a comprehensive and integrated diagnostic approach. While morphological assessment serves as the foundation, it must be complemented by targeted immunocytochemistry, EBER in situ hybridization, flow cytometry, and molecular assays to accurately differentiate among overlapping entities. Awareness of the key cytologic features, combined with the judicious application of ancillary studies, is essential for achieving an accurate diagnosis and guiding appropriate clinical management.

## Data Availability

No datasets were generated or analysed during the current study.

## Abbreviations

BL: Burkitt lymphoma
DLBCL: Diffuse large B-cell lymphoma
EBER: EBV-encoded RNA
EBV + nT/NKCL: EBV-positive nodal T and NK cell lymphoma
ENKTL: Extranodal NK/T-cell lymphoma
LDH: Lactate dehydrogenase
LGL: Large granular lymphocyte
PCR: Polymerase chain reaction
PBL: Plasmablastic lymphoma
PEL: Primary effusion lymphoma
PET-CT: Positron emission tomography-computed tomography
TCR: T-cell receptor
TIA-1: T-cell intracellular antigen-1
T/NKCL: T/NK-cell lymphoma
*TRG*: T-cell receptor gamma chain gene
WHO-HAEM5: The 5th edition of WHO Classification of Haematolymphoid Tumours
